# The risk factors analysis and establishment of an early warning model for healthcare-associated infections after pediatric cardiac surgery: A STROBE-compliant observational study

**DOI:** 10.1097/MD.0000000000023324

**Published:** 2020-12-04

**Authors:** Lihui Meng, Jiachen Li, Yan He, Ying Xiong, Jingming Li, Jing Wang, Ying Shi, Yinglong Liu

**Affiliations:** a Pediatric Cardiac Center, Department of Cardiac Surgery; b Health-care Associated Infection Management Office, Beijing Anzhen Hospital, Capital Medical University, Beijing, China.

**Keywords:** early warning model, health-care associated infections, pediatric cardiac surgery, risk factors

## Abstract

The aim of this study was to identify the main risk factors for health-care-associated infections (HAIs) following cardiac surgery and to establish an effective early warning model for HAIs to enable intervention in an earlier stage.

In total, 2227 patients, including 222 patients with postoperative diagnosis of HAIs and 2005 patients with no-HAIs, were continuously enrolled in Beijing Anzhen Hospital, Beijing, China. Propensity score matching was used and 222 matched pairs were created. The risk factors were analyzed with the methods of univariate and multivariate logistic regression. The receiver operating characteristic (ROC) curve was used to test the accuracy of the HAIs early warning model.

After propensity score matching, operation time, clamping time, intubation time, urinary catheter time, central venous catheter time, ≥3 blood transfusions, re-endotracheal intubation, length of hospital stay, and length of intensive care unit stay, still showed significant differences between the 2 groups. After logistic model analysis, the independent risk factors for HAIs were medium to high complexity, intubation time, urinary catheter time, and central venous catheter time. The ROC showed the area under curve was 0.985 (confidence interval: 0.975–0.996). When the probability was 0.529, the model had the highest prediction rate, the corresponding sensitivity was 0.946, and the specificity was 0.968.

According to the results, the early warning model containing medium to high complexity, intubation time, urinary catheter time, and central venous catheter time enables more accurate predictions and can be used to guide early intervention after pediatric cardiac surgery.

## 1 Introductions

Patients after pediatric cardiac surgery often have an elevated rate of postoperative health-care-associated infections (HAIs) because of severe primary disease, low immunity, multiple invasive operations and postoperative therapies, catheter-associated bloodstream infections (CLABSIs), and surgical site infections (SSIs).^[1]^ Some data have shown that the rate of HAIs after surgery for congenital heart disease (CHD) is between 2.7% and 8%.^[2,3]^ These complications could cause prolonged hospitalizations, indicate a worse prognosis, and increase medical expenses.^[4,5]^ Although both the treatment concept and means of preventing infections have been improved,^[6]^ most preventive and treatment experience regarding HAIs stems from adults, and few articles have reported a systematic and effective HAI forecasting method for children after pediatric cardiac surgery.

The aim of this study was to identify the incidence, aetiology, and main risk factors of HAIs following cardiac surgery in a population with congenital heart disease and to establish an effective early warning model for HAIs to enable intervention at an earlier stage.

## 2 Materials and methods

### 2.1 Definition of HAIs

According to the recommendations of the Centers for Disease Control and Prevention, an HAI is defined as an infection arising during hospitalization or within specified times after discharge that was neither clinically manifest nor in incubation at the time of admission.^[7]^ The postoperative nosocomial infections investigated in this study included but were not limited to the following: ventilator-associated pneumonia (VAP), bloodstream infections, CLABSIs, SSIs, catheter-associated urinary tract infections, antibiotic-associated diarrhoea, chest infections, abdominal infections, postoperative infective endocarditis, and skin soft-tissue infections; infections of the upper respiratory tract were not included.

### 2.2 Setting and study population

The study was carried out at the Department of Pediatric Cardiac Surgery and the Department of Cardiac Intensive Care Unit in Beijing Anzhen Hospital, Beijing, China. All patients were continuously enrolled from January 1, 2016 to November 30, 2017. The exclusion criteria included: clearly diagnosed genopathy, infective endocarditis, perioperative infections of other positions, patients>14 years old, cases of data loss, clinical death within 24 hours after operation. According to the definition of HAIs and criteria above, 2227 children, including 222 HAIs patients and 2005 no-HAIs patients, were enrolled. Capital Medical University Affiliated Anzhen Hospital's Ethics Committee approved the study protocol (Institutional Review Board File 2016021) and consent was obtained from the patients or their relatives.

### 2.3 Operative strategy and procedure

It is well established that there are many operative methods to address a variety of CHDs. In these circumstances, to standardize the operative methods performed in our patients, we implemented the Risk Adjustment in Congenital Heart Surgery (RACHS-1) score.^[8]^

### 2.4 Data collection

**Figure 1:**
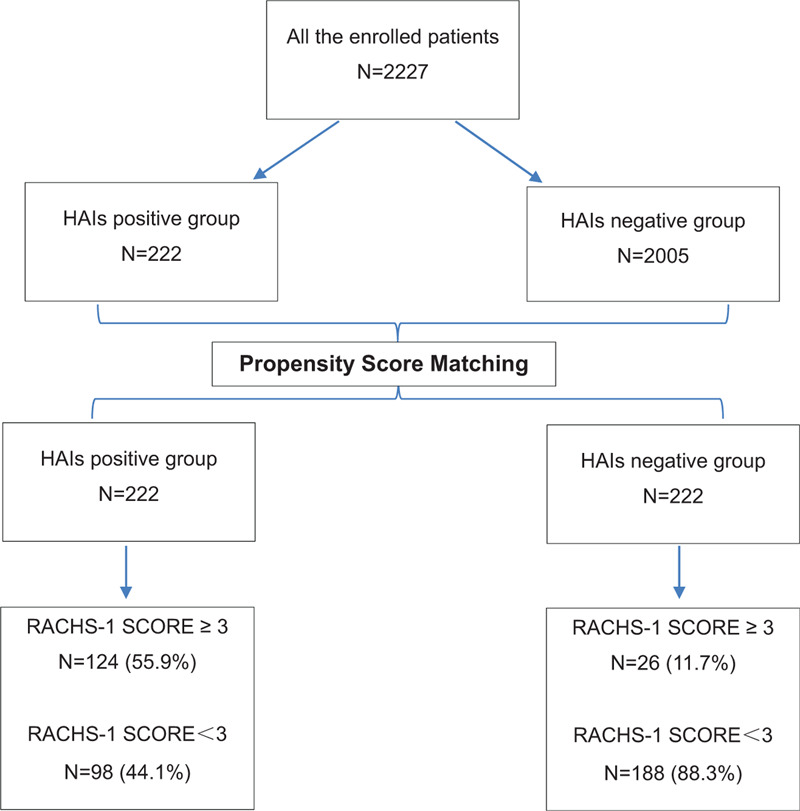
Study protocol. HAIs = health-care-associated infections, RACHS-1 SCORE= Risk Adjustment in Congenital Heart Surgery score.

Data from all patients were collected on a specific form concerning the patient's personal and medical history, symptoms and signs, type of heart disease, and laboratory and imaging findings. The preoperative specific factors evaluated included age, gender, body length, weight, admission hemoglobin level, admission serum total protein level, admission serum albumin level, admission serum prealbumin level, ejection fraction value, the type of heart disease. The intraoperative factors included the category of RACHS-1, the length of the operation, the length of the clamped aorta. The postoperative factors included the use and duration of mechanical ventilation, the use and duration of a urinary catheter, the use and duration of a central venous catheter, the use and duration of antibiotics, the number of blood transfusions, the performance of other invasive operations, the total duration of the hospital stay, and the total duration of the intensive care unit (ICU) stay. Other invasive operations included thoracic drainage tube replacement, re-endotracheal intubation, peritoneal dialysis tube, reoperation, delayed sternal closure, extracorporeal membrane oxygenation, and permanent pacemaker implantation. The study protocol is shown in Figure 1.

### 2.5 Statistical analysis

Data are presented as the means ± standard deviations for continuous data with a normal distribution, as the medians (25th percentile, 75th percentile) for continuous data with a non-normal distribution, or as numbers and percentages for categorical variables.

Propensity scores were calculated with the following preoperative variables: age, sex, weight, body length, admission hemoglobin level, admission serum total protein level, admission serum albumin level, and admission serum prealbumin level. Matching was performed using a Greedy 5-to-1 Digit-Matching algorithm. After propensity score matching, 222 matched pairs were created (Table 1). In propensity-matched patients, univariate analyses were carried out using paired *t* tests or the Wilcoxon signed-rank test for continuous variables and the Pearson *χ*^2^ test for categoric variables. Previous reports suggested standard criteria for the propensity score-matching method,^[9]^ and we followed those criteria in this analysis.

**Table 1:** Preoperative, intraoperative, and postoperative information.

	Propensity score-matched patients		
Variables	HAIs (N=222)	No-HAIs (N=222)	*P* value
Baseline characteristics			
^∗^Age, yrs	0.74 (0.35, 1.00)	1.00 (0.40, 2.00)	*P* = .925
^†^Male	122 (55.0%)	115 (51.8%)	*P* = .505
Body length, cm	74.21±24.62	75.76±20.35	*P* = .471
^∗^Weight, kg	7.35 (4.98, 10.00)	7.60 (5.20, 10.70)	*P* = .384
Admission hemoglobin, g/L	13.20±2.80	13.12±1.40	*P* = .722
Admission serum total protein, g/L	59.89±7.69	59.17±6.24	*P* = .279
Admission serum albumin, g/L	41.79±4.00	42.47±6.03	*P* = .160
^∗^Admission serum prealbumin, g/L	0.13 (0.11, 0.15)	0.13 (0.11, 0.16)	*P* = .343
Surgical information			
^†^RACHS-1 SCORE≥3	124 (55.9%)	26 (11.7%)	*P* < .001
Operation time, min	252.05±109.28	155.01±50.76	*P* < .001
Clamping time, min	78.69±42.28	40.27±22.44	*P* < .001
Postoperative information			
^∗^Intubation time, h	146.50 (81.50, 273.75)	28.50 (17.00, 65.00)	*P* < .001
^∗^Urinary catheter time, d	7.0 (4.0, 13.0)	5.0 (3.0, 7.0)	*P* < .001
Central venous catheter time, d	17.93±9.63	5.76±2.67	*P* < .001
^†^Blood transfusion≥3 times	115 (51.8%)	22 (9.9%)	*P* < .001
^†^Other invasive operations	63 (28.4%)	6 (2.7%)	*P* < .001
Thoracic drainage tube replacement	13	1	*P* = .817
Re-endotracheal intubation	10	3	*P* = .041
Peritoneal dialysis tube	12	0	–
Reoperation	15	2	*P* = .605
Delayed sternal closure	4	0	–
Extracorporeal membrane oxygenation	6	0	–
Permanent pacemaker implantation	3	0	–
Hospital stay, d	35.51±20.50	13.35±6.42	*P* < .001
ICU stay, d	15.44±10.42	7.40±2.80	*P* < .001

Values are mean ± SD, ^∗^median (25th percentile, 75th percentile) and ^†^ n (%). In group HAIs, there were 36 patients who underwent 63 “other invasive operations.” In group no-HAIs, there were 9 patients who underwent 9 “other invasive operations.” HAIs = health-care associated infections, ICU = intensive care unit, RACHS-1 SCORE = Risk Adjustment in Congenital Heart Surgery score, SD = standard deviation.

Univariate and multivariate logistic regression were used to identify independent risk factors (Table 2). Receiver operating characteristic (ROC) curve analysis was used to predict the effectiveness of the early warning model (Table 3). Statistical significance was defined as *P*<.05 with 2-tailed distributions. All statistical analyses were performed with SPSS version 22.0 software (SPSS Inc, Chicago, IL).

**Table 2:** Univariate logistic regression model assessing the risks for HAIs in patients undergoing cardiac surgery for CHD.

Risk for HAIs	β(B)	Standard error	Odds ratio	95% confidence interval		*P* value
Medium to high complexity	1.098	0.579	2.999	0.963	9.334	.058
Operation time	0.006	0.004	1.006	0.998	1.015	.146
Clamping time	0.010	0.010	1.010	0.992	1.029	.282
Intubation time	0.038	0.006	1.039	1.026	1.052	<.001
Urinary catheter time	−0.767	0.139	0.464	0.354	0.609	<.001
Central venous catheter time	0.556	0.096	1.743	1.443	2.105	<.001
Blood transfusion≥3 times	0.873	0.684	2.394	0.626	9.157	.202

RACHS-1 score of 1 and 2 as low complexity, RACHS-1 score of 3 as medium complexity, RACHS-1 score of 4 and 5 as high complexity. CHD = congenital heart disease, HAIs = health-care-associated infections, RACHS-1 = Risk Adjustment in Congenital Heart Surgery score.

**Table 3:** Multivariate logistic regression model assessing the risks for HAIs in patients undergoing cardiac surgery for CHD.

Adjusted risk for HAIs	β(B)	Standard error	Odds ratio	95% confidence interval		*P* value
Medium to high complexity	1.957	0.590	5.079	2.632	16.165	<.001
Intubation time	0.043	0.006	1.049	0.958	1.069	<.001
Urinary catheter time	0.774	0.132	2.169	1.678	2.804	<.001
Central venous catheter time	−0.606	0.094	0.545	0.454	0.655	<.001

CHD = congenital heart disease, HAIs = health-care-associated infections, RACHS-1 = Risk Adjustment in Congenital Heart Surgery score.

## 3 Results

### 3.1 Preoperative, surgical, and postoperative information of propensity score-matched patients

The preoperative data of the propensity-matched patients are listed in Table 1. The matched pairs were well balanced for all known covariates, including age, sex, weight, body length, admission hemoglobin level, admission serum total protein level, admission serum albumin level, admission serum prealbumin level, and ejection fraction. The intraoperative and postoperative data of the propensity score matched patients are also listed in Table 1. After propensity score matching, there were 124 (55.9%) patients who underwent cardiac surgery with RACHS-1 scores ≥3 in the treatment group (HAI group), while in the control group (no-HAI) group, only 26 (11.7%) patients underwent operations with RACHS-1 scores ≥3. The intraoperative data demonstrated that the operation time and clamping time were significantly longer in the HAI group than in the no-HAI group. Postoperative data, including intubation time, urinary catheter time, central venous catheter time, ≥3 blood transfusions, re-endotracheal intubation, the length of hospital stay and the length of ICU stay, still showed significant differences between the 2 groups. For some other invasive operations including the use of peritoneal dialysis tube or extracorporeal membrane oxygenation, delayed sternal closure, and permanent pacemaker implantation, there were no patients who underwent those operations in the no-HAIs group, so the statistic results were not received.

### 3.2 The risks for HAIs in patients undergoing cardiac surgery for CHD

We put the variables that showed significant differences between 2 groups in Table 1 into the univariate logistic regression model to analyze the risk for HAIs in patients undergoing cardiac surgery; the result was demonstrated in Table 2. It should be pointed out that *Hospital stay* and *ICU stay* were excluded from the logistic analysis, because these 2 variables were not the risk factors for HAIs but caused by HAIs. According to RACHS-1 score, a RACHS-1 score of 4 or more is called high complexity, a RACHS-1 of 3 is called medium complexity. *Medium to high complexity* was put together as an independent risk factor, compared with operations with a RACHS-1 of 1 or 2 in our research. Multivariate analysis for the variables after univariate analysis demonstrated that the independent risk factors for HAIs after cardiac surgery were medium to high complexity (*P*<.001), intubation time *(P<.001)*, urinary catheter time (*P<.001*), and central venous catheter time *(P<.001)* (Table 3). On the contrary, some variables such as blood transfusion≥3 times were not regarded as the independent risk factors.

### 3.3 Early warning performance for HAIs

**Figure 2:**
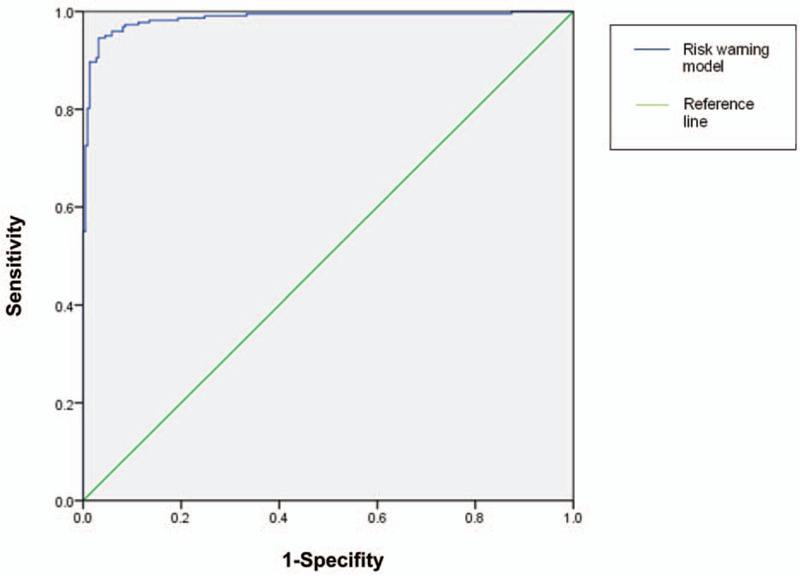
ROC curve of the warning model for patients with high risk of HAIs. HAIs = health-care-associated infections, ROC = receiver operating characteristic.

We established an early warning model to predict the risk of HAIs after pediatric cardiac surgery for children. We use the data from the HAI-positive group to test the accuracy of the model; the area under curve of the ROC curve is 0.985 (confidence interval: 0.975–0.996) (Fig. 2). When the probability was 0.529, the model had the highest prediction rate, and the Youden index was 0.914. When we take 0.529 as the risk probability value of the validation group (≥0.529 identified as infected, <0.529 identified as not infected), the corresponding sensitivity is 0.946, the specificity is 0.968, the positive predictive value is 94.6%, and the negative predictive value is 96.8% (Table 4).

**Table 4:** The result of verification for the risk warning model McNemar test, *P* = .359; Kappa = 0.914, *t* = 19.273, *P* < .001.

		Confirmed diagnosis	
Model identification results		HAIs (N = 222)	No-HAIs (N = 222)
Prediction	Positive	210	7
	Negative	12	215

HAIs = health-care-associated infections.

## 4 Discussion

Our study confirms that major infection after congenital heart surgery is a complication with significant sequelae. Using a large patient population from a single center, we identified risk factors for healthcare-associated infections and created a clinical tool that can be used preoperatively to estimate a patient's infection risk. We validated the model internally, showing that it has good discrimination.

### 4.1 Research status

It has been reported that the incidence rate of HAIs is 2.7% to 8% worldwide.^[2,3]^ A Japanese hospital studied 526 young patients (under 18 years old) after cardiac surgery from January 2013 to December 2015; 81 patients were diagnosed with postoperative HAIs. The infective rate was 15.4%, including 30 blood-borne infections (5.7%), 30 operative site infections (5.7%), 13 urinary tract infections (2.5%), and 8 pulmonary infections (1.5%).^[10]^ Another retrospective study of 634 CHD patients from the USA reported that 38 patients were diagnosed with HAIs 90 days after surgery, including 19 blood-borne infections (3.0%), 9 operative site infections (1.4%), 6 infective endocarditis (0.95%), and 4 ventilator-related pulmonary infections (0.6%); the total infection rate was 5.99%.^[11]^ Many studies have confirmed^[12–15]^ that young age, underweight, long operation time, long clamping time, long mechanical ventilation time, and long ICU stay are risk factors for HAIs, but the results were inconsistent to varying degrees in quantitative risk indicator studies. A study in neonates from the USA reported that the risk factors for HAIs also included central venous catheter indwelling time >14 days and >5 postoperative blood transfusions.^[13]^ In addition, the American Society of Anesthesiologists (ASA) score, ICU stay >48 hours,^[2]^ the difficulty of the operation (RACHS-1 score), and cardiopulmonary bypass (CPB) time ≥200 minutes are closely related to the development of postoperative HAIs.^[14]^ Another study from Georgia, USA reported that young patients <1 year old, emergency surgery, reoperation for any reason, reintubation, and a history of hospitalization in other hospitals within the past 3 months have also been shown to be risk factors for HAIs.^[15]^

Some device-related infections have been paid particular attention by researchers investigating HAIs after operations for CHD; these infections include VAP, SSIs, and CLABSIs. Costello et al^[16]^ reported that young patients <1 year old, CPB time >105 minutes, aortic cross-clamp time >85 minutes, >3 postoperative blood transfusions, hospitalization time before operation >48 hours could be independent risk factors for SSIs and could guide clinical practice. Other studies reported in foreign countries, including Canada and the United States, indicated that the ASA score, mechanical ventilation time, length of ICU stay, length of postoperative, white blood count before operation and the first day after operation, and duration of vasoactive drug use should also receive attention when attempting to prevent SSIs after an operation.^[17]^ The influence of the use of perioperative antibiotics on SSIs has always been controversial; some of the related studies confirmed that antimicrobial prophylaxis over 48 hours after the operation did not reduce the incidence of SSIs but rather increased the risk of infection, including the rates of drug-resistant *Bacillus* infections and *Clostridium difficile* infections.^[18,19]^ In addition, it has been broadly accepted that the incidences of VAP and CLABSIs are closely related to long durations of invasive catheterization and multiple catheter implantation.

### 4.2 The use of propensity score matching

Based on the studies described above, certain preoperative conditions could be high-risk factors for postoperative infections, including low birth weight, poor nutritional status, and preoperative pulmonary infections. Moreover, age, body length, and sex may also be influential factors affecting the incidence of postoperative infections. Under the circumstances, propensity scores were used because we mainly wanted to explore how the intraoperative and postoperative operations influenced HAIs. In fact, we first used all the 2227 patients (including 222 HAIs patients and 2005 non-HAIs patients) enrolled to analyze the differences between 2 groups. We found that some preoperative items could have significant differences including age (0.74 (0.35, 1.00) years versus 1.00 (1.00, 3.00) years, *P<.001*), body length (74.21±24.62 cm vs 91.52±34.69 cm, *P<.001*) and weight (7.35 (4.98, 10.00) kg versus 9.60 (7.00, 14.00) kg, *P<.001*, the data above were not demonstrated in the article). Therefore, we used the method of propensity score matching to effectively remove confounding factors, a number of typical preoperative items including age, sex, weight, body length, admission hemoglobin level, admission serum total protein level, admission serum albumin level, and admission serum prealbumin level were calculated in the method. Admittedly, some preoperative items such as age, weight, and preoperative nutritional status could surely affect the risk of postoperative infection, but we mainly discussed the medical procedures related factors that could generate HAIs in this research.

### 4.3 The use of RACHS-1

RACHS-1 score is a very important variable for our research. It allows a variety of pediatric operation procedures to be measured in a uniform way. As many articles did,^[20]^ we regarded operations with RACHS-1 score≥3 as medium to high complexity, RACHS-1 score<3 as low complexity. We could clearly find out that *Medium to high complexity* may be the most crucial risk factor for postoperative HAIs, compared with *Intubation time*, *Urinary catheter time,* and *Central venous catheter time* from Tables 2 and 3. It means that when patients receiving surgeries of medium to high complexity, the predictive risk for HAIs could increase more than 4 times compared with patients receiving low complexity surgeries. By contrast, the other 2 important perioperative risk factors, *Operation time* and *Clamping time*, showed no significant difference between 2 groups. It means that extracorporeal circulation technique, myocardial, and respiratory protection technique have made most pediatric cardiac ratios implement safely, while it is the scale and range of operation and patients’ systematic state, especially whether cyanosis preoperatively, that most impact the HAIs.

### 4.4 The early warning model

Finally, we created a predictive model for HAIs based on the above results. The monitoring indexes included medium to high complexity, intubation time, urinary catheter time, and central venous catheter time. It is also very important to reasonably determine the weighting of these early-warning indicators and warning thresholds. Our research finally determined the weights of the indicators according to their beta coefficients as follows: Medium to high complexity, 5.079; intubation time, 1.049; urinary catheter time, 2.169; central venous catheter time, 0.545. The ROC curve provides the Youden's index (YDI, sensitivity+ specifity-1) value for each point on the coordinate; the best threshold is usually determined by the optimal truncation point corresponding to the maximum value of the YDI. In this study, when the optimal truncation point was 0.529, the YDI was the maximum value of 0.914. Compared with other warning models,^[20]^ the model created and validated in this study could have an important clinical impact because it provides a more accurate estimate of an individual patient's risk for major infectious complications. Identification of these high-risk patients is useful in the early stage of the postoperative period by helping parents and providers know what obstacles may lie ahead. In addition, these identified high-risk patients may be targeted for future clinical trials and interventions to reduce this complication of cardiac surgery.

### 4.5 When confronting HAIs

Apart from the complexity of the operations, some factors related to postoperative invasive procedures including intubation time, urinary catheter time, and central venous catheter time are also closely related to postoperative HAIs. This result reminds us of some important concepts in clinical practice: First, remove all unnecessary invasive devices as soon as possible; Second, sufficiently manage all invasive operations, including maintaining aseptic conditions during device implantation, performing adequate postoperative clinical care, and avoiding repeated punctures; Third, it should be a routine for most institutes to monitor the tip culture as an indicator for HAIs. Admittedly, some other invasive operations mentioned in this article, including re-endotracheal intubation, peritoneal dialysis tube, reoperation, extracorporeal membrane oxygenation, and so on, were proved to be independent risk factors for hospital infection. But after propensity matching, the sample size of these operations was rather small in no-HAIs group, we could not make valid comparison between the 2 groups. Another distinctly important factor for hospital infection must be the application of antibiotics. In our research, all of the surgeries were performed with prophylactic antibiotics under anesthesia, primary antibiotic would be continued for 3 days after surgery, and may undergo a combination therapy of 2 antibiotics during stay in ICU, depending on infection control. All antibiotic changes and upgrades are conducted in accordance with the guidelines.

## 5 Conclusion

Medium to high complexity, intubation time, urinary catheter time, and central venous catheter time are independent risk factors for HAIs after cardiac surgery. The early warning model containing these 4 factors for HAIs enables more accurate predictions and can be used to guide early intervention after pediatric cardiac surgery.

### 5.1 Study limitations

We adjusted for as many potentially confounding variables as possible by performing propensity score matching and multivariable analysis. But there may be selection bias in the patients so that some sample size was too small, such as re-endotracheal intubation and the use of extracorporeal membrane oxygenation. These invasive operations have been proved to be specific risk factors for HAIs, but could not be verified in our research.

## Author contributions

XXXX.

## References

[1] Geroulanos S Rosmarakis ES Falagas ME Frequency, characteristics, and predictors of microbiologically documented nosocomial infections after cardiac surgery. Eur J Cardio thorac Surg 2006;29:456–60. 10.1016/j.ejcts.2005.12.035 16481186

[2] Levy I Ovadia B Erez E Nosocomial infections after cardiac surgery in infants and children: incidence and risk factors. Hosp Infect 2003;53:111–6. 10.1053/jhin.2002.1359 12586569

[3] Sohn AH Schwartz JM Yang KY Risk factors and risk adjustment for surgical site infections in pediatric cardiothoracic surgery patients. Am J Infect Control 2010;38:706–10. 20605267 10.1016/j.ajic.2010.03.009

[4] Li LY Wang SQ Economic effects of nosocomial infections in cardiac surgery. J Hosp Infect 1990;4:339–41. 10.1016/0195-6701(90)90006-a 1980506

[5] Martone WJ Nichols RL Recognition, prevention, surveillance, and management of surgical site infections: introduction to the problem and symposium overview. Clin Infect Dis 2001;33: suppl 2: S67–8. 11486301 10.1086/321859

[6] Harbarth S Samore MH Lichtenberg D Prolonged antibiotic prophylaxis after cardiovascular surgery and its effect on surgical-site infections and its effect on surgical-site infections and antimicrobial resistance. Circulation 2000;101:2916–21. 10869263 10.1161/01.cir.101.25.2916

[7] Lazar HL How important is glycemic control during coronary artery bypass? Adv Surg 2012;46:219–35. 22873042 10.1016/j.yasu.2012.03.007

[8] Jenkins KJ Gauvreau K Newburger JW Consensus-based method for risk adjustment for surgery for congenital heart disease. J Thorac Cardiovasc Surg 2002;123:110–8. 11782764 10.1067/mtc.2002.119064

[9] McMurry TL Hu Y Blackstone EH Propensity scores: methods, considerations, and applications in the Journal of Thoracic and Cardiovascular Surgery. J Thorac Cardiovasc Surg 2015;150:14–9. 25963441 10.1016/j.jtcvs.2015.03.057

[10] Hatachi T Tachibana K Inata Y Risk factors for healthcare-associated infections after pediatric cardiac surgery. Pediatr Crit Care Med 2018;19:237–44. 29319633 10.1097/PCC.0000000000001445 PMC5841862

[11] Turcotte RF Brozovich A Corda R Health care-associated infections in children after cardiac surgery. Pediatr Cardiol 2014;35:1448–55. 24996642 10.1007/s00246-014-0953-z

[12] García H Cervantes-Luna B González-Cabello H Risk factors for nosocomial infections after cardiac surgery in newborns with congenital heart disease. Pediatr Neonatol 2017;23:9570–2. 10.1016/j.pedneo.2017.11.014 29248382

[13] Sahu MK1 Siddharth CB1 Devagouru V Hospital-acquired Infection: prevalence and outcome in infants undergoing open heart surgery in the present era. Indian J Crit Care Med 2017;21:281–6. 28584431 10.4103/ijccm.IJCCM_62_17 PMC5455021

[14] Barriga J Cerda J Abarca K Nosocomial infections after cardiac surgery in infants and children with congenital heart disease. Rev Chilena Infectol 2014;31:16–20. 24740769 10.4067/S0716-10182014000100002

[15] Lomtadze M Chkhaidze M Mgeladze E Incidence and risk factors of nosocomial infections after cardiac surgery in Georgian population with congenital heart diseases. Georgian Med News 2010;178:7–16. 20157198

[16] Costello JM Graham DA Morrow DF Risk factors for surgical site infection after cardiac surgery in children. Ann Thorac Surg 2010;89:1833–41. 20494036 10.1016/j.athoracsur.2009.08.081

[17] Sochet AA Cartron AM Nyhan A Surgical site infection after pediatric cardiothoracic surgery. World J Pediatr Congenit Heart Surg 2017;8:7–12. 28033082 10.1177/2150135116674467

[18] Edwards FH Engelman RM Houck P Society of Thoracic Surgeons practice guideline series: antibiotic prophylaxis in cardiac surgery, part I: duration. Ann Thorac Surg 2006;81:397–404. 16368422 10.1016/j.athoracsur.2005.06.034

[19] Carignan A Allard C Pepin J Risk of Clostridium difficile infection after perioperative antibacterial prophylaxis before and during an outbreak of infection due to a hypervirulent strain. Clin Infect DisV 46 2008;1838–43. 10.1086/588291 18462108

[20] Kansy A Jacobs JP Pastuszko A Major infection after pediatric cardiac surgery: a risk estimation model. Ann Thorac Surg 2012;94:2091–5. 23040826 10.1016/j.athoracsur.2012.07.079

